# Barium enema findings in total colonic aganglionosis: a single-center, retrospective study

**DOI:** 10.1186/s12887-020-02403-3

**Published:** 2020-10-31

**Authors:** Jiayu Yan, Jihang Sun, Rongchang Wu, Sarah Siyin Tan, Yongwei Chen, Yun Peng, Yajun Chen

**Affiliations:** 1 Department of General Surgery, Beijing Children’s Hospital, National Center of Children’s Health, Capital Medical University, Beijing, China; 2 Department of Radiology, Beijing Children’s Hospital, National Center of Children’s Health, Capital Medical University, Beijing, China; 3 Department of Neonatal Surgery, Beijing Children’s Hospital, National Center of Children’s Health, Capital Medical University, Beijing, China; 4 Beijing, PR China

**Keywords:** Total colonic aganglionosis, Preoperative, Barium enema, Radiographic sign, Diagnosis

## Abstract

**Background:**

Preoperative diagnosis of total colonic aganglionosis is important for the rational choice of treatment. The present study aimed to evaluate the diagnostic performance of radiographic signs on preoperative barium enema in patients with total colonic aganglionosis.

**Methods:**

Forty-four patients [41 (3-659) days] with total colonic aganglionosis, including 17 neonatal patients, who received preoperative barium enema at Beijing Children’s Hospital, from January 2007 to December 2019 were included. All radiographs were retrospectively restudied by 2 pediatric radiologists to ascertain radiographic signs including rectosigmoid index, transition zone, irregular contraction, gas-filled small bowel, microcolon, question-mark-shaped colon and ileocecal valve reflux. Kappa test was performed to assess the accuracy and consistency of the radiographic signs.

**Results:**

The 2 radiologists showed slight agreement for gas-filled small bowel, microcolon and rectosigmoid index, fair agreement for transition zone and irregular contraction, and moderate agreement for question-mark-shaped colon and ileocecal valve reflux (Kappa values, 0.043, 0.075, 0.103, 0.244, 0.397, 0.458 and 0.545, respectively). In neonatal patients, the 2 radiologists showed moderate agreement for ileocecal valve reflux and substantial agreement for question-mark-shaped colon (Kappa values, 0.469 and 0.667, respectively). In non-neonatal patients, the 2 radiologists showed substantial agreement for ileocecal valve reflux (Kappa value, 0.628). In 36 patients with total colonic aganglionosis extending to the ileum, the accuracies of question-mark-shaped colon, ileocecal valve reflux and the combination of both were 47%, 53%, and 75%, respectively, in one radiologist and 53%, 50% and 72%, respectively, in the other radiologist.

**Conclusions:**

Ileocecal valve reflux is a relatively reliable radiographic sign for diagnosing total colonic aganglionosis and could improve the diagnostic accuracy upon combination with question-mark-shaped colon.

## Background

Total colonic aganglionosis (TCA) is a rare disease characterized by the absence of enteric ganglion cells in the colon with or without extension to the ileum that occurs in approximately 3–15% of patients with Hirschsprung’s disease (HD) [1–5]. With advancements in surgical techniques and meticulous management, the mortality rate and bowel function of patients with TCA have improved [6]. The important problem in TCA is not surgical management but rather its prompt diagnosis, which decreases the rate of misdiagnosis and allows patients to receive treatment timely to adequately handle neonatal symptoms by conservative or surgical treatment, which decreases preoperative mortality [2, 4, 7–9]. In patients with “segmental” HD, contrast enema can help distinguish HD from alternate etiologies prior to rectal biopsy, but rectal biopsy is the standard approach for preoperative diagnosis [10]. However, in cases with aganglionosis of the entire colon and occasionally the small bowel, contrast enema is the only non-invasive method available for the investigation of the entire colonic morphology and assisted diagnosis of TCA before intraoperative multiple full-thickness punch biopsy [7].

Many radiographic features that can be observed on contrast enema have been described as useful signs for diagnosing “segmental” HD. These features mainly include 24-h delayed radiography, as described by Ehrenpreis in 1945; the transition zone and irregular contraction, as described by Swenson in 1949; the rectosigmoid index, as described by Pochaczevsky in 1975; the microcolon, as described by Berdon in 1964; and gas-filled small bowel, which indicates intestinal obstruction with gas-fluid levels and gaseous distention of bowel loops [11–15]. However, very few studies have assessed their usefulness in patients with TCA. Some radiographic features, such as question-mark-shaped colon and ileocecal valve reflux, are considered typical radiographic features in patients with TCA [16–18]. However, all above-described radiographic features were identified as being quite difficult to diagnose on TCA in one of the largest studies, which included 17 patients with TCA [7].

Several studies have reported that water-soluble contrast agents, such as iothalamate meglumine, can be applied to the diagnosis of TCA and have sensitivities and specificities for detection equivalent to those of barium contrast [7, 16, 19]. However, some problems are associated with water-soluble contrast, including the risk of dehydration and a lack of research [20]. Barium enema remains the most widely used method for diagnosing TCA in most centers [19, 21].

Therefore, the purpose of this study was to evaluate the diagnostic performance of preoperative barium enema and radiographic signs in TCA by retrospectively reviewing all complete radiographs in patients who were confirmed to have TCA by postoperative pathological examination at one of the largest pediatric centers in China.

## Methods

### Study patients

**Fig. 1:**
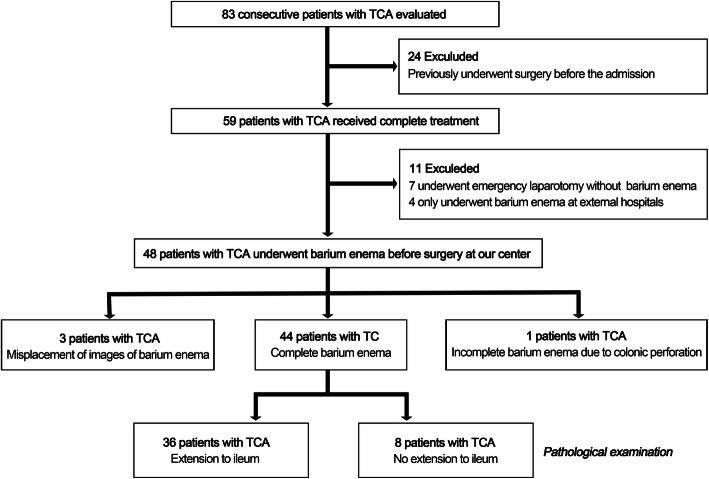
Flow diagram of the patient enrollment process in this study

This retrospective study was approved by the ethics committee of Beijing Children’s Hospital, National Center of Children’s Health, China (2020-k-11). The requirement for informed consent was waived by the institutional review board. By searching the medical records system database, we retrospectively identified patients who were assigned the diagnosis code “total colonic aganglionosis” according to the International Classification of Diseases, ninth revision (ICD-10; code Q43.106) at our center from January 2007 to December 2019. The records of 83 clinical cases with pathologically confirmed TCA with or without extension to ileum were obtained (Fig. 1). Individuals were excluded from the study population for any of the following reasons: underwent surgical treatment before admission (*n* = 24), underwent emergency laparotomy without preoperative barium enema due to severe clinical symptoms following conservative treatment (*n* = 7), received barium enema at only external hospitals before surgical treatment at ours (*n* = 4), and incomplete images of barium enema due to misplacement (*n* = 3) or failure to barium enema due to colonic perforation (*n* = 1). All patients included in the study were confirmed to have TCA with involvement of 30 cm or less of the terminal ileum by pathological examination [22, 23]. All clinical data were abstracted to determine demographics, age at time of barium enema whenever possible, the original radiology report of barium enema, and pathological results.

### Contrast enema techniques

All contrast enemas were performed with water-diluted barium in an unprepared colon by the same radiologic team at our center. The general technique was to place a catheter (without a rectal balloon) by taping it to the skin. The catheter was then held by the parents who were dressed in lead. The catheter was inserted approximately one inch into the rectum while the patient was in a lateral position. Barium medium was then slowly instilled using a syringe. The colon was examined in anteroposterior and lateral projections. At our center, 24 h-delayed radiographs are no longer routinely recorded given their low sensitivity and specificity. All radiographs were reviewed by an experienced pediatric radiologist, and an original radiology report was obtained prior to the surgical treatment.

### Radiographic signs and analysis

Available original preoperative barium enema radiographs were collected by a pediatric surgeon (with 3 years of experience) who had a good understanding of the clinical details and pathological results. A recent barium enema performed at our center before the initial surgery was collected because many patients had undergone barium enema at external hospitals before referral to our center. The radiographs were independently and randomly analyzed by 2 experienced attending radiologists (radiologist 1with more than 15 years of experience with a focus in gastrointestinal research; radiologist 2 with 6 years of experience). The reviewers were unaware of the clinical details and original radiology report.

**Fig. 2:**
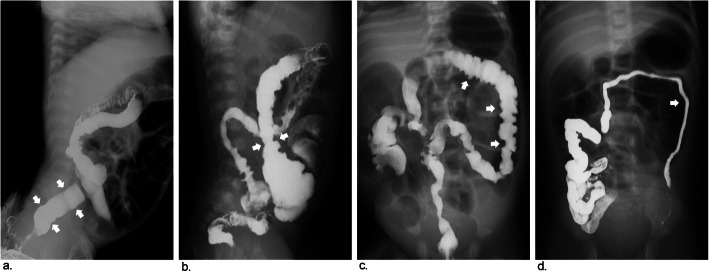
Images show common and typical radiographic signs of preoperative BEs in patients with HD. **a** Rectosigmoid index ≤ 1, obtained by dividing the widest diameter of the rectum by the widest diameter of the sigmoid loop *(arrows)*. **b** Transitional zone, regarded as the site of obvious caliber change from nondilated to dilated bowel *(arrows)*. **c** Irregular contraction, resulted from denervation hyperspasticity of the distal segment with a sawtooth configuration *(arrows)*. **d** Microcolon, a small-caliber colon with the largest diameter less than 1 cm *(arrow)*

**Fig. 3:**
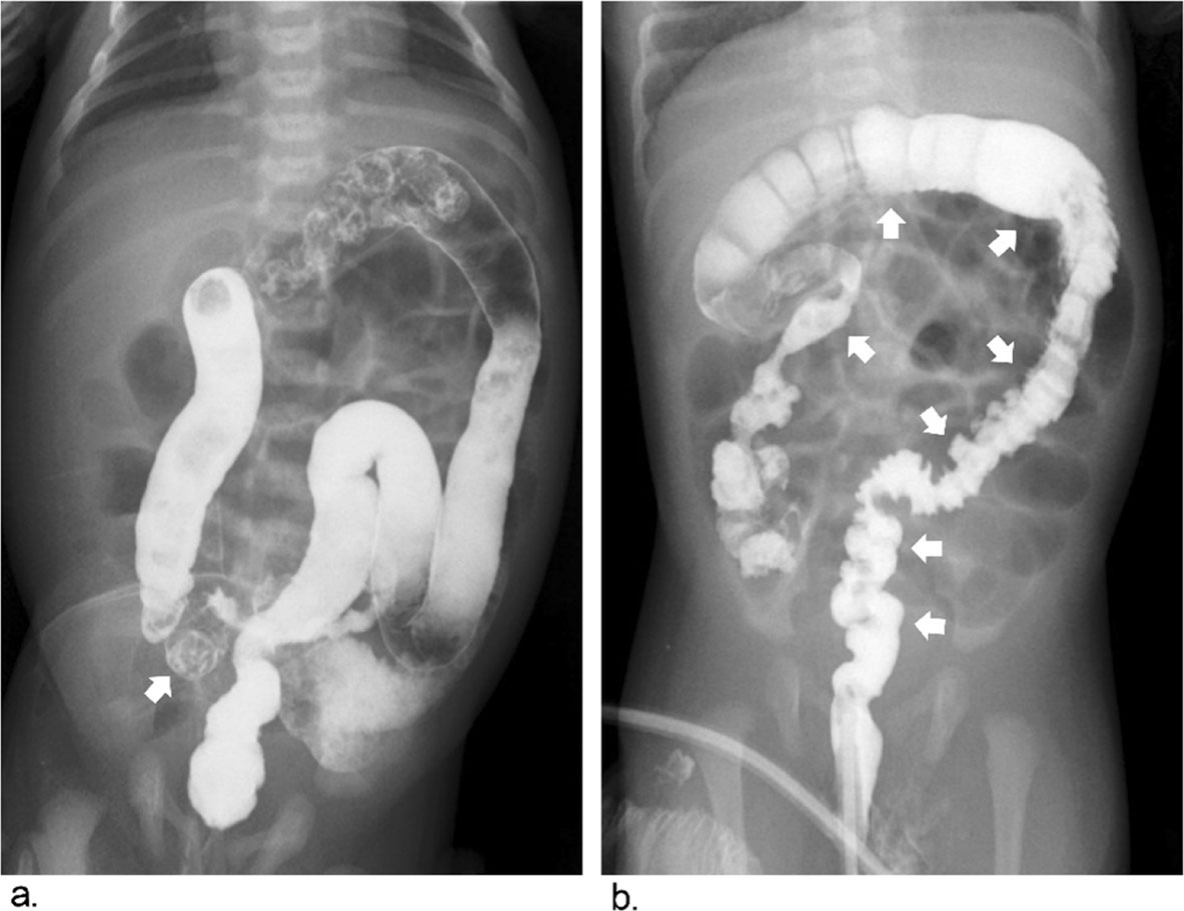
Images show common and typical radiographic signs of preoperative BEs in patients with TCA. **a** Ileocecal valve reflux, reflux of contrast material into the terminal ileum *(arrow)*. **b** Question-mark-shape colon, described as a rounded and shortened contour of hepatic and splenic flexures with short appearing colon *(arrows)*

**Fig. 4:**
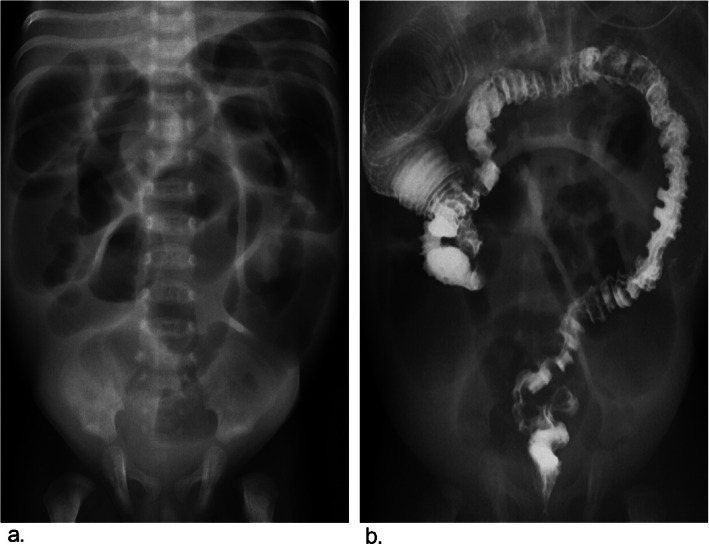
A patient with TCA has all common and typical radiographic signs of preoperative BEs: rectosigmoid index ≤ 1, transition zone, irregular contraction, gas-filled small bowel, a microcolon, question-mark-shape colon and ileocecal valve reflux

The criteria for radiographic signs were discussed and assigned prior to the study. The following 7 radiographic signs were evaluated in the barium enema of each patient [6, 16, 19]. Five radiographic signs were used to diagnose “segmental” HD: rectosigmoid index (obtained by dividing the widest diameter of the rectum by the widest diameter of the sigmoid loop when the colon was fully distended by contrast medium and the normal RI is > 1), transition zone (regarded as a site of obvious caliber change from nondilated to dilated bowel), irregular contraction (denervation hyperspasticity of the distal segment with a sawtooth configuration), gas-filled small bowel (intestinal obstruction with gas-fluid levels and gaseous distention of bowel loops) and microcolon (a small-caliber colon with a largest diameter less than 1 cm). The following two radiographic signs were associated with TCA: question-mark-shaped colon (described as a rounded and shortened contour of hepatic and splenic flexures with the ascending colon) and ileocecal valve reflux (reflux of contrast material into the terminal ileum) (Figs. 2, 3 and 4).

### Statistical methods

The results of barium enema analysis were compared with the results of postoperative pathological examination as a percentage value. Values for accuracy were determined for rectosigmoid index, transition zone, irregular contraction, gas-filled small bowel, microcolon, question-mark-shaped colon and ileocecal valve reflux. The calculation of interexaminer agreement for the results of radiographic signs was evaluated using Kappa test. Kappa values of 0-0.20, 0.21–0.40, 0.41–0.60, 0.61–0.80, 0.81–0.99, and 1.00 were considered to represent slight, fair, moderate, substantial, excellent, and absolute agreement, respectively. *P*-values less than 0.05 defined statistical significance. Statistical calculations were performed using SPSS, version 22.0 (IBM, Armonk, NY).

## Results

### Patient demographics

The study included 44 patients with available data for a preoperative barium enema completed at our center. They were aged 3-659 (median age: 41) days old, and 17 of the patients were newborns (**≤** 30 days after birth). Thirty (30/44, 68%) patients had an original radiology report of suspected HD (12/44, 27%) and TCA (18/44, 41%), and these patients had a median age of 41 (3-521) days. Thirty-six patients and 8 patients had a definite diagnosis of TCA with and without extension to the ileum, respectively, based on postoperative pathological results.

### Evaluation of radiographic sign diagnostic capacity

The diagnostic capacity of barium enema for rectosigmoid index **≤** 1 determination, transition zone, irregular contraction, gas-filled small bowel, microcolon, question-mark-shaped colon and ileocecal valve reflux identification in patients with TCA was analyzed both separately and combined by 2 radiologists (Table 1).

**Table 1:** Accuracy and consistency of radiographic signs between the radiologists

Radiographic signs	Accuracy		Consistency		
	Radiologist 1	Radiologist 2	Number	Kappa value	*P* value
HD radiographic signs					
Rectosigmoid index ≤ 1	38/44 (86)	17/44 (39)	16/44 (36)	0.103	0.234
Transition zone	12/44 (27)	26/44 (59)	10/44 (23)	0.244	0.045
Irregular contraction	13/44 (30)	25/44 (57)	12/44 (27)	0.397	0.002
Gas-filled small bowel	34/44 (77)	43/44 (98)	33/44 (75)	0.043	0.012
Microcolon	39/44 (89)	30/44 (68)	26/44 (61)	0.075	0.547
TCA radiographic signs					
Question-mark-shape colon	21/44 (48)	25/44 (57)	17/44 (36)	0.458	0.002
Ileocecal valve reflux	23/44 (52)	23/44 (52)	18/44 (41)	0.545	0.000

Abbreviations: *TCA *total colonic aganglionosis; *HD *Hirschsprung’s disease

For the radiographic signs used to diagnose “segmental” HD, radiologist 1 recorded rectosigmoid index **≤** 1 in 38 patients (38/44, 86%) and identified transition zone, irregular contraction, gas-filled small bowel and microcolon in 12 (12/44, 27%), 13 (13/44, 30%), 34 (34/44, 77%) and 39 (39/44, 89%) patients, respectively. Radiologist 2 recorded rectosigmoid index **≤** 1 in 17 patients (17/44, 39%) and identified transition zone, irregular contraction, gas-filled small bowel and microcolon, in 26 (26/44, 59%), 25 (25/44, 57%), 43 (43/44, 98%) and 30 (30/44, 68%) patients, respectively. The 2 radiologists showed slight agreement for gas-filled small bowel, microcolon and rectosigmoid index **≤** 1, and fair agreement for transition zone and irregular contraction, with Kappa values as 0.043 (*P* = 0.012), 0.075 (*P* = 0.547), 0.103 (*P* = 0.234), 0.244 (*P* = 0.045), and 0.397 (*P* = 0.002), respectively.

Regarding radiographic signs associated with TCA, radiologist 1 identified question-mark-shaped colon and ileocecal valve reflux in 21 (21/44, 48%) and 23 (23/44, 52%) patients, respectively. Radiologist 2 identified question-mark-shaped colon and ileocecal valve reflux in 25 (25/44, 57%) and 23 (23/44, 52%) patients, respectively. The 2 radiologists showed moderate agreement for question-mark-shaped colon and ileocecal valve reflux, with Kappa values as 0.458 (*P* = 0.002) and 0.545 (*P* = 0.000), respectively.

### Evaluation of question-mark-shaped colon and ileocecal valve reflux in neonatal and non-neonatal patients

Our analysis of diagnostic value of question-mark-shaped colon and ileocecal valve reflux identification in neonatal and non-neonatal patients is shown in Table 2. Of the 17 neonatal patients, radiologist 1 identified question-mark-shaped colon and ileocecal valve reflux in each 9 patients (9/17, 53%). Radiologist 2 determined question-mark-shaped colon and ileocecal valve reflux in each 8 patients (8/17, 47%). The 2 radiologists showed moderate agreement for ileocecal valve reflux and substantial agreement for question-mark-shaped colon, with Kappa values as 0.469 (*P* = 0.040) and 0.667 (*P* = 0.004), respectively. In 27 non-neonatal patients, radiologist 1 identified question-mark-shaped colon and ileocecal valve reflux in 12 (12/27, 44%) and 14 (14/27, 52%) patients, respectively, while radiologist 2 identified question-mark-shaped colon and ileocecal valve reflux in 17 (17/27, 63%) and 15 (15/27, 56%) patients, respectively. The 2 radiologists showed fair agreement on question-mark-shaped colon and substantial agreement on ileocecal valve reflux, with Kappa values as 0.352 (*P* = 0.050) and 0.628 (*P* = 0.001), respectively.

**Table 2:** Accuracy and consistency of question-mark-shape colon and ileocecal valve reflux between the radiologists

Radiographic	Neonatal					Non-neonatal				
	Accuracy		Consistency			Accuracy		Consistency		
	Radiologist 1^a^	Radiologist 2^b^	Number	Kappa value	*P* value	Radiologist 1^c^	Radiologist 2^d^	Number	Kappa value	*P* value
Question-mark-shape colon	9/17 (53)	8/17 (47)	7/17 (41)	0.667	0.004	12/27 (44)	17/27 (63)	10/27 (36)	0.352	0.050
Ileocecal valve reflux	9/17 (53)	8/17 (47)	6/17 (35)	0.469	0.040	14/27 (52)	15/27 (56)	12/27 (41)	0.628	0.001

^a^In neonatal patients with TCA, radiologist 1 identified question-mark-shape colon and ileocecal valve reflux in 5 (5/17, 29%) same patients

^b^In neonatal patients with TCA, radiologist 2 identified question-mark-shape colon and ileocecal valve reflux in 4 (4/17, 24%) same patients

^c^ In non-neonatal patients with TCA, radiologist 1 identified question-mark-shape colon and ileocecal valve reflux in 5 (5/27, 19%) same patients

^d^ In non-neonatal patients with TCA, radiologist 2 identified question-mark-shape colon and ileocecal valve reflux in 4 (4/27, 15%) same patients

### Evaluation of question-mark-shaped colon and ileocecal valve reflux in patients with TCA extension to the ileum

In the subset of 36 patients with TCA extension to the ileum, the diagnostic capacity and combined diagnostic accuracy of question-mark-shaped colon and ileocecal valve reflux identification are shown in Table 3. Radiologist 1 identified question-mark-shaped colon and ileocecal valve reflux in 17 (17/36, 47%) and 19 (19/36, 53%) patients, while radiologist 2 identified question-mark-shaped colon and ileocecal valve reflux in 19 (19/36, 53%) and 18 (18/36, 50%) patients, respectively. The 2 radiologists showed poor agreement for question-mark-shaped colon and ileocecal valve reflux, with Kappa values as 0.003 (P = 0.985) and 0.167 (P = 0.317), respectively. The use of both question-mark-shaped colon and ileocecal valve reflux combined was a good predictor of the presence of TCA extension to the ileum (75% and 72% accuracy, respectively).

**Table 3:** Accuracy and consistency of question-mark-shape colon and ileocecal valve reflux for patients with TCA extension to ileum

	Accuracy		Consistency			Combination
	Question-mark-shape colon^a^	Ileocecal valve reflux^b^	Number	Kappa value	*P* value	
Radiologist 1	17/36 (47)	19/36 (53)	9/36 (25)	0.003	0.985	27/36 (75)
Radiologist 2	19/36 (53)	18/36 (50)	11/36 (31)	0.167	0.317	26/36 (72)

Abbreviations: *TCA *total colonic aganglionosis

^a^Two radiologists identified question-mark-shape colon in 11 (11/36, 31%) same patients

^b^Two radiologists identified ileocecal valve reflux in 10 (10/36, 28%) same patients

## Discussion

Our study is the largest case series of patients with TCA at a single center to evaluate the diagnostic performance of preoperative barium enema [7, 24]. The major advantages of patient enrollment in our study are as follows. First, enrollment spanned from 2007 to 2019, during which radiologic contrast enema was well recognized and specifically applied for HD [25, 26]. Second, only patients who did not undergo any surgical treatment prior to barium enema were observed in our study. This potentially reduced confounding errors due to surgical interference by the bowel. Finally, all examinations were conducted in a single center, and the operating supplies, techniques and diagnostic algorithms were consistent for all patients. More importantly, all available radiographic signs regarding HD and TCA were included in our study to evaluate the overall abdominal condition of the patient.

We evaluated the diagnostic accuracy of preoperative barium enema and demonstrated that the radiographic signs used to diagnose “segmental” HD, including rectosigmoid index, transition zone, irregular contraction, gas-filled small bowel and microcolon, were not reliable for diagnosing TCA. Previous studies indicated that rectosigmoid index ≤ 1 and transition zone exhibited potential value for diagnosing “segmental” HD with sensitivity values from 68–86% and 63–94%, respectively. In addition, the combination of rectosigmoid index ≤ 1 and transition zone was more useful for screening for HD [27, 28]. However, in patients with TCA, the barium enema does not always present with these characters. Based on our results, radiologist 1 obtained a rectosigmoid index ≤ 1 in 34 patients (34/44, 86%) with TCA, which was twice the value obtained by radiologist 2 (17/44, 39%). When the whole colon and rectum are aganglionic and spasmodic, rectal ampulla may be difficult to identify in barium enema, even for experienced radiologists. Different radiologists may choose different radiographs to record rectosigmoid index ≤ 1 in a patient, which would influence judgment. Transition zone is regarded as an effective radiographic sign to identify the extent of aganglionic bowel in “segmental” HD and could therefore facilitate surgical planning [21]. In our study, the two radiologists noted these occurrences in 12 (12/44, 27%) and 26 (26/44, 59%) patients, and both of these values were higher than that reported in a previous study of TCA (2/17, 12%) [7]. However, transition zone was still mostly found in the transverse and the ascending colon, which is consistent with previous studies demonstrating that the pathologic extent of aganglionic bowel is more proximal than the site of the transition zone [7, 29]. This finding may be related to the patients receiving conservative treatment, which creates high pressure in the proximal aganglionic colon and can mislead surgical planning [30]. Rectosigmoid index ≤ 1 and transition zone appear to be more accurate in “segmental” HD compared with TCA [30, 31]. Irregular contraction was previously found in approximately 50% of patients with HD and TCA, a finding that was confirmed by our results but showed fair agreement among radiologists [15, 25]. Gas-filled small bowel and microcolon are often simultaneously identified at the time of barium examination in most TCA patients, both neonatal and non-neonatal, who suffered from chronic defecation difficulties [15]. Our results consistent with a recent study by Shan Zheng et al., but good diagnostic consistency was not noted among different radiologists [32]. For microcolon, there is no reported standard measurement available for different ages, particularly in newborns, given the lack of consistency in the X-ray findings, thus limiting the application of diagnosis, which is consistent with our study [7, 33].

Question-mark-shaped colon was first described by Sane and Girdany in 1973 but only a small number of patients with TCA exhibit typical question-mark-shaped colon [16, 17]. Ileocecal valve reflux was first systematically described by Chandler in 1970 but was found to be a nonspecific finding in newborns, in which it also depended on the amount and pressure of the rectal contrast agent used during the enema [7, 18]. Although question-mark-shaped colon and ileocecal valve reflux were not particularly sensitive, the 2 radiologists in our study showed moderate agreement in the accuracy of question-mark-shaped colon and ileocecal valve reflux in patients with TCA, indicating the potential diagnostic performance. Therefore, we further explored the value of the question-mark-shaped colon and ileocecal valve reflux identification in neonatal and non-neonatal patients, even in patients with TCA extension to the ileum. Our results showed that ileocecal valve reflux was a reliable radiographic sign in preoperative barium enema for both neonatal and non-neonatal patients with TCA. Although poor consistency was noted in diagnosis between question-mark-shaped colon and ileocecal valve reflux identification between individual radiologists, the combination of question-mark-shaped colon and ileocecal valve reflux identification in both radiologists could increase accuracy by up to 75%. This finding may help clinicians perform accurate intraoperative multiple full-thickness punch biopsy and develop appropriate medical treatments [12, 14].

Our study had some limitations. First, because our study design was retrospective, there is a potential risk of selection bias. Second, these analyses were performed using collected barium enema images, which prevented a dynamic analysis and potentially influenced the radiological interpretation. Third, a control group comprising children without HD diagnosis or children with HD without TCA was not included. Therefore, we suggest that this issue should be further studied. Finally, the number of patients included in our study was limited to 44 patients. Multicenter studies about preoperative barium enema involving more patients with TCA are expected to be performed in the future.

## Conclusions

Ileocecal valve reflux is a relatively reliable radiographic sign for diagnosing total colonic aganglionosis and could improve the diagnostic accuracy upon combination with question-mark-shaped colon.

## Data Availability

All data generated or analyzed during this study are included in this published article.

## Abbreviations

TCA: Total colonic aganglionosis
HD: Hirschsprung's disease
